# Debranching thoracic endovascular aortic repair for distal aortic arch aneurysm in elderly patients aged over 75 years old

**DOI:** 10.1186/s13019-020-1047-z

**Published:** 2020-01-10

**Authors:** Suguru Shiraya, Yoshinobu Nakamura, Shingo Harada, Yuichiro Kishimoto, Takeshi Onohara, Yuki Otsuki, Tomohiro Kurashiki, Hiromu Horie, Motonobu Nishimura

**Affiliations:** Division of Cardiovascular Surgery, Tottori University Faculty of Medicine, 36-1, Nishi-cho, Yonago, Tottori, 683-8504 Japan

**Keywords:** Debranching TEVAR, Thoracic aortic aneurysm in the elderly, Distal arch aneurysm

## Abstract

**Background:**

We examined the outcome of debranching thoracic endovascular aortic repair (d-TEVAR) without sternotomy for distal aortic arch aneurysm in patients aged ≥75 years.

**Methods:**

Patients who underwent d-TEVAR or TAR for aortic arch aneurysm between 2008 and 2015 at our hospital and aged ≥75 years were included. Age, sex, left ventricular ejection fraction, preoperative creatinine level, diabetes, cerebrovascular disease, and chronic obstructive pulmonary disease were matched using PS.

**Results:**

Among 74 patients (d-TEVAR: 51, TAR: 23), 17 patients in each group were matched. No difference in surgical outcome was detected between the d-TEVAR and TAR groups, including 30-day death (0% vs. 0%), hospital death (5.8% vs. 0%: *p* = 0.31) and incidence of cerebral infarction (5.8% vs. 7.6%: *p* = 0.27) as well as the long-term outcomes of 5-year survival (92.8% vs. 74.8%: *p* = 0.30) and 5-year aorta-related event-free rate (88.2% vs. 100%: *p* = 0.15). Average duration of ICU stay (1.3 ± 1.1 days vs. 5.6 ± 1.3 days: *p* = 0.025) and hospital stay (16.5 ± 5.2 days vs. 37.7 ± 19.6 days: *p* = 0.017) were significantly shorter in the d-TEVAR group.

**Conclusion:**

Our results indicated that d-TEVAR is less invasive without affecting long-term outcome up to 5 years. Although the number of the patients included in the study was small, debranching TEVAR could be one of the treatments of the choice in the elderly, especially with comorbidities.

## Introduction

Surgery for aortic arch aneurysm has been associated with high mortality and serious complications. However, recent advances in surgical procedures have remarkably decreased the mortality rates and the incidence of postoperative complications [1, 2]. Combined with advances in surgical procedures, an aging of society has meant that the number of surgery for the elderly has been increasing. While the safety of surgery has been improving, advanced age has been reported to be a risk factor for surgery. Thus, careful examination of the treatment strategy is required for the treatment of thoracic aortic aneurysm in the elderly [3]. In recent years, treatment of aneurysm has entered a new era after the endografts for endovascular treatment became commercially available. The safety of the endovascular treatment for the descending thoracic aortic aneurysm and abdominal aortic aneurysm has been established, and a favorable long-term outcome has been reported [4, 5]. However, the safety and long-term outcomes of the endovascular treatment for aortic arch aneurysm remain controversial as it requires the additional procedures, such as cervical debranching. Since 2008, we have applied debranching thoracic endovascular aortic repair (d-TEVAR) mainly for selected patients. Essentially, we selected patients with aortic arch aneurysm arising distal to left carotid artery (distal arch aneurysm) and aged over 75 years old. One of the problems of endovascular treatment for aortic aneurysms is the increased aneurysm-related mortality after 10 years as shown in EVAR trial 1 [6]. We decided this cut-off criteria of 75 years of age, because average life expectancy was about 10 years at the age of 75 in Japan [7]. Even in elderly patients over 75 years old with distal arch aneurysm, total arch replacement (TAR) had to be selected, when enough proximal landing zone could not be secured or landing zone was considered to be shaggy. In this study, the early and mid-term outcome of d-TEVAR was evaluated and compared with that of total arch replacement through median sternotomy (TAR) as treatment procedures for distal aortic arch aneurysm in elderly individuals aged ≥75 years.

## Methods

### Patients

Patients who had aortic arch aneurysm arising distal to left carotid artery (distal arch aneurysm) and underwent d-TEVAR or TAR for degenerative aortic arch aneurysm between 2008 and 2015 at our hospital and aged ≥75 years were included in this study. Patients were indicated for surgery if exhibited a thoracic aortic aneurysm of ≥60 mm. In principle, debranching TEVAR for aortic arch aneurysm without sternotomy was performed, if feasible, after introducing d-TEVAR in 2010, whereas TAR was performed in patients with aneurysms extending to near the brachiocephalic artery so that sufficient proximal landing zone was not assured. Patients who received endovascular treatment with sternotomy or with chimney technique were not included. A closed-chest technique was adopted for all debranching TEVAR, and bypass to the cervical branch or simple closing of the left subclavian artery was simultaneously performed. TAR was performed for patients in whom closed-chest debranching TEVAR is anatomically difficult to perform.

Patients who received endovascular aortic repair with cervical debranching technique or simple closing of the left subclavian artery when treating aortic arch aneurysm were included in the debranching TEVAR group (d-TEVAR), and patients who received TAR were included in the TAR group.

This study was carried out in accordance with principles outlined in the Declaration of Helsinki. It was conducted after obtaining approval from the ethics committee of Tottori University Faculty of Medicine.

### Procedures of endovascular treatment

**Fig. 1:**
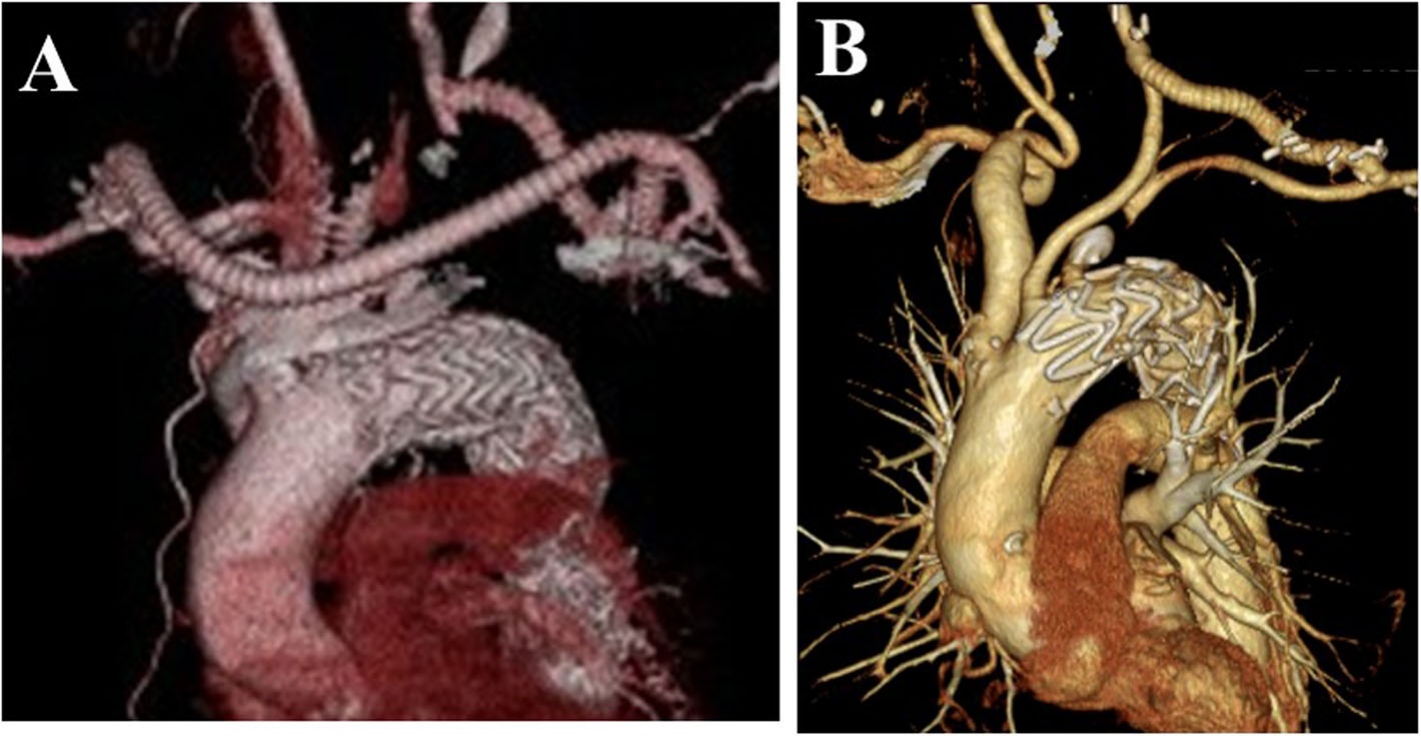
Representative image of 3-D computed tomography after debranching TEVAR. **a** Zone 1 landing. Right axillary artery - left common carotid artery - left axillary artery bypass. **b** Zone 2 landing. Left common carotid artery – left axillary artery bypass and embolization of the origin of left subclavian artery

Treatment strategy was determined according to Zone classification, in principle. The proximal side of the endograft should be able to be fixed in zone 1 or 2 in patients in the d-TEVAR group. At least 2 cm of the landing zone to the proximal side was ensured. For graft landing in zone 1, the left common carotid artery (LCCA) and the left axillary artery (LAA) were bypassed from the right axillary artery (RAA). As a basic policy, the LCCA was tied at the proximal to the anastomosis of the bypass and the origin of the left subclavian artery was coiled after bypass (Fig. 1a). For graft landing in zone 2, LCCA-LAA bypass, or RAA-LAA crossover bypass was performed. The origin of the left subclavian artery was also coiled after bypass in this case as a basic policy (Fig. 1b).

### Procedures of total arch replacement

A median sternotomy approach was employed for all patients. Cardiopulmonary bypass was established with venous drainage from the superior and inferior vena cava and the blood return to the right axillary artery and the either side of the femoral artery. Core body temperature was cooled to ≤25 °C and total arch replacement was performed with quadrifurcated graft under selective cerebral perfusion and open distal technique.

### Postoperative follow-up and data analysis

Patients were periodically followed-up as outpatients following surgery, and contrast enhanced or plain computed tomography was performed once a year during the follow-up period. If patients failed to visit hospital, survival was confirmed via telephone.

Permanent neurological deficits (PND) were defined as the presence of deficits at the time of hospital discharge. Transient neurological deficits (TND) were defined as the deficits that recovered by hospital discharge.

As endpoints, 30-day death, hospital death, duration in ICU, incidence of PND, spinal cord disorder, duration in hospital, transfer to a rehabilitation hospital, time until home discharge, requirement for postoperative tubal feeding, cumulative survival rate, and aorta-related event-free rate were evaluated.

### Statistical analysis

All numerical data are shown as averages ± standard deviation (SD). All data were analyzed using PASW Statistics ver. 24 (IBM SPSS Inc., Chicago, USA). The unpaired t test was used for comparisons of continuous variables. We utilized Kaplan-Meier curves and the log-rank test to compare the endpoints. Differences with a *P* value < 0.05 were considered statistically significant.

Owing to the non-randomized nature of the study, and considering for significant differences in baseline characteristics, propensity-score matching was used to control for potential confounders of the treatment outcome relationship. Propensity scores were calculated using logistic regression with surgical procedure as the dependent variable. The propensity score included 10 variables, including age, sex, hypertension, chronic obstructive pulmonary disease (FEV1.0% < 70%) (COPD), diabetes (treatment with insulin or oral hypoglycemic agents), history of coronary disease (history of percutaneous coronary intervention or coronary bypass), hemodialysis, previous sternotomy, history of cerebrovascular disease, and preoperative creatinine level. For each patient of debraching TEVAR group, a propensity score-matched patient of TAR group was selected (1:1) using the one-to-one nearest neighbor method and no replacement.

## Results

**Fig. 2:**
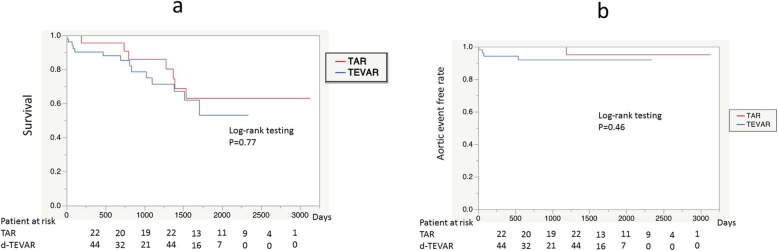
Long-term results before propensity-score matching. **a** Survival rate after treatment of each group. **b** Aortic event free rate after treatment of each group

The total number of patients was 74, which included 51 patients in the d-TEVAR group and 23 patients in the TAR group. Patients in the d-TEVAR group were significantly older and significantly more patients were complicated with COPD in the TEVAR group (Table 1). No difference was observed in the surgical outcome of 30-day death (1.9% vs. 0%: *p* = 0.49), hospital death (5.8% vs. 0%: *p* = 0.13), and the incidence of PND (3.9% vs. 4.3%: *p* = 0.68) between the d-TEVAR and TAR groups. Average duration in ICU (2.1 ± 2.6 days vs. 4.8 ± 6.8 days: *p* = 0.016) and the average duration in hospital (17.8 ± 19.2 days vs. 35.6 ± 18.5 days: *p* = 0.0004) variables were significantly increased in the TAR group, whereas transfer to a rehabilitation hospital for recovery of activities of daily living (ADL) was observed more often in the TAR group (17.6% vs. 26.0%: *p* = 0.41). The time until home discharge (median) was significantly increased in the TAR group (34 days) compared with that in the d-TEVAR group (13 days) (*p* = 0.016) (Table 2). No significant difference was observed in the mid-term outcomes of 5-year survival (53.0% vs. 63.0%: *p* = 0.47) (Fig. 2a) and 5-year aorta-related event-free rate (91.8% vs. 95.0%: *p* = 0.46) between the d-TEVAR and TAR groups (Fig. 2b).

**Table 1:**
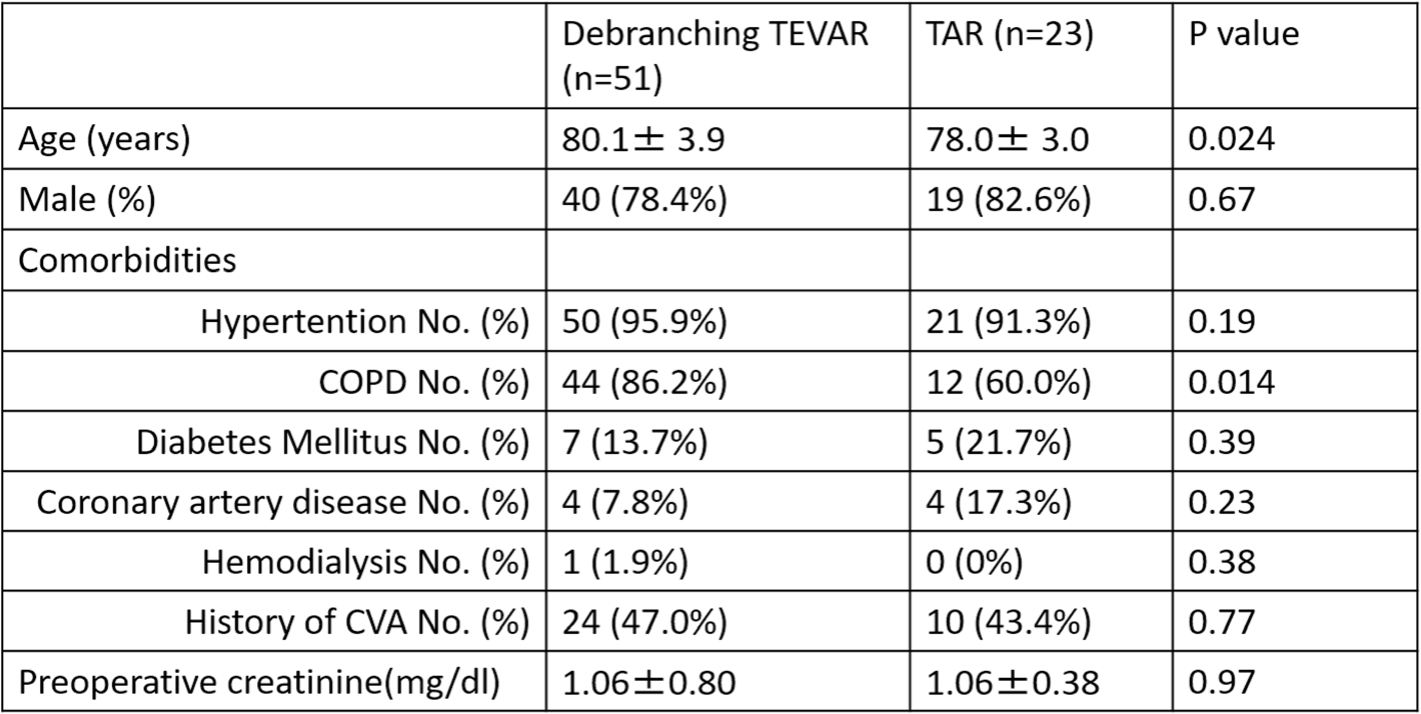
Patients characteristics

**Table 2:** Outcome after treatment

	Debranching TEVAR (*n* = 51)	TAR (*n* = 23)	*P* value
30 days mortality. No. (%)	1 (1.9)	0 (0)	0.49
In-hospital death No. (%)	3 (5.9)	0 (0)	0.13
PND No. (%)	2 (3.9)	1 (4.3)	0.68
TND No. (%)	1 (1.9)	1 (4.3)	0.53
Spinal cord injury No. (%)	1 (1.9)	0 (0)	0.38
ICU stay (median)	2.1 ± 2.6 (1)	4.8 ± 6.8 (3)	0.016
Hospital Stay (median)	17.8 ± 19.2 (13)	35.6 ± 18.5 (35)	0.0004
Transfer to another hospital No. (%)	9 (17.6)	6 (26.0)	0.41
Total Hospitalization (median)	26.8 ± 30.3 (13)	47.2 ± 38.6 (34)	0.016

During matching of age, sex, hypertension, COPD, history of diabetes, coronary disease, hemodialysis, previous sternotomy, and history of cerebrovascular disease, preoperative creatinine level using PS, 17 patients each of the d-TEVAR group (*n* = 51) and the TAR group (*n* = 23) were matched. Following matching, patient backgrounds exhibited favorable matching (Table 3).

**Table 3:** Patients characteristics after propensity-score matching

	Debranching TEVAR (*n* = 17)	TAR (*n* = 17)	*P* value
Age (years)	76.9 ± 2.0	77.4 ± 2.2	0.52
Male (%)	14 (82.4%)	15 (88.2%)	0.62
Comorbidities			
Hypertention No. (%)	17 (100%)	15 (88.2%)	0.089
COPD No. (%)	12 (70.5%)	11 (64.7%)	0.71
Diabetes Mellitus No. (%)	2 (11.8%)	4 (23.5%)	0.36
Coronary artery disease No. (%)	4 (23.5%)	5 (29.4%)	0.69
HemodialysisNo. (%)	0 (0%)	0 (0%)	
History of CVA No. (%)	5 (29.4%)	6 (35.3%)	0.71
Preoperative creatinine (mg/dl)	0.88 ± 0.31	1.11 ± 0.41	0.07

**Fig. 3:**
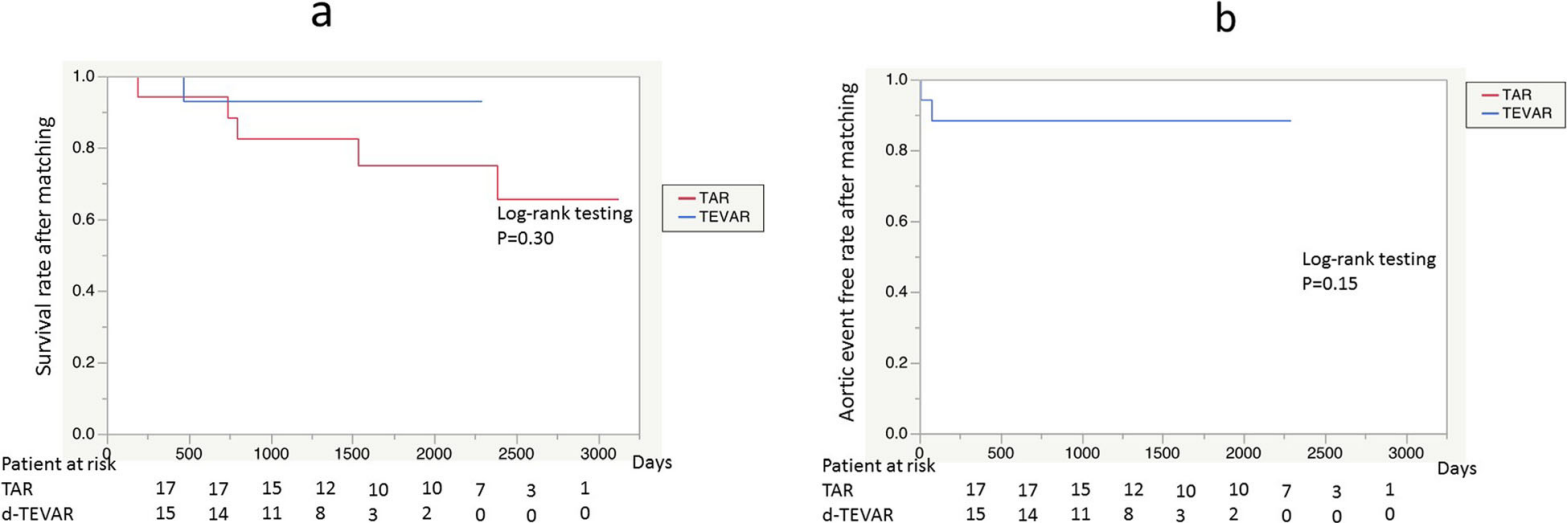
Long-term results after propensity-score matching. **a** Survival rate of each group after propensity-score matching. **b** Aortic event free rate of each group after propensity-score matching

No difference was observed in the surgical outcome of 30-day death (0% vs. 0%), hospital death (5.8% vs. 0%: *p* = 0.23), and the incidence of PND (5.8% vs. 5.8%: *p* = 0.76) between the d-TEVAR and TAR groups. The average duration in ICU (1.6 ± 1.5 days vs. 5.6 ± 7.8 days: *p* = 0.025) and the average duration in hospital (16.5 ± 16.2 days vs. 37.7 ± 19.6 days) were significantly increased in the TAR group (*p* = 0.017), whereas transfer to a rehabilitation hospital for the recovery of ADL was observed significantly more often in the TAR group (5.8% vs. 29.4%: *p* = 0.041). The time until home discharge (median) was significantly increased in the TAR group (34 days) compared with that in the d-TEVAR group (12 days) (*p* = 0.011) (Table 4). No significant difference was observed in the long-term outcomes of 5-year survival (92.8% vs. 74.8%: *p* = 0.30) (Fig. 3a) and 5-year aorta-related event-free rate (88.2% vs. 100%: *p* = 0.15) (Fig. 3b) between the d-TEVAR and TAR groups.

**Table 4:** Outcome after propensity-score matching

	Debranching TEVAR (*n* = 17)	TAR (*n* = 17)	*P* value
30 days mortality. No. (%)	0 (0)	0 (0)	
In-hospital death No. (%)	1 (5.8)	0 (0)	0.23
PND No. (%)	1 (5.8)	1 (5.8)	0.76
TND No. (%)	0 (0)	1 (5.8)	0.50
Spinal cord injury No. (%)	0 (0)	0 (0)	
ICU stay (median)	1.6 ± 1.5 (1)	5.6 ± 7.8 (3)	0.025
Hospital Stay (median)	16.5 ± 16.2 (12)	37.7 ± 19.7 (34)	0.0017
Transfer to another hospital No. (%)	1 (5.8)	5 (29.4)	0.041
Total Hospital Stay (median)	20.5 ± 23.9 (12)	52.6 ± 43.2 (34)	0.011

## Discussion

In this study, the incidence of perioperative death and cerebral infarction was low after debranching TEVAR and TAR, and treatment of aortic arch aneurysm was performed with relative safety in elderly individuals aged ≥75 years. Our indication of surgery was the aneurysm exceeding 60 mm in diameter, which was a little bit larger than the current ESC guideline. In this study, we increased the threshold due to the advanced age of the patients. In bibliographic consideration of surgical treatment for the elderly, Khaladj et al. examined 501 patients who underwent surgery using anterograde selective cerebral perfusion for the ascending aortic aneurysm and aortic arch aneurysm, and reported that age was an independent risk factor for early death [3]. Okita et al. reported that operative mortality and complication rates were significantly higher in patients in their 70s and 80s, thus, thoracic aorta surgery appears to be a risk for the elderly [8]. However, according to a more recent report that showed a mortality rate of ~ 1% in elective surgery, risk of surgery itself has been significantly decreased [9]. Hiraoka et al. reported that age was not a risk factor in aortic arch aneurysm [10]. Based on these findings, surgery for thoracic aortic aneurysm in elderly individuals aged ≤80 has been accepted [11]. In our hospital, operative mortality and hospital mortality, and the incidence of PND were relatively low (even in the elderly), thus, it was indicated that surgery may be performed safely in this population. However, PND was reported as an independent predictor of postoperative mortality in both d-TEVAR and TAR groups [12, 13]; this aspect probably reflects the influence of atherosclerotic changes in the aortic arch and neck vessels, rather than procedural differences between the two groups.

However, the duration of ICU and hospital stay in the elderly population evaluated in this study were significantly shorter in the debranching TEVAR group, as compared with those in the TAR group, and the number of days required for home discharge was also significantly shorter in the debranching TEVAR group. Therefore, these results showed that more time is required for the recovery of ADL in the elderly in the TAR group, despite the lower incidence of perioperative death and cerebral infarction. According to Lohse et al., postoperative quality of life is significantly reduced if the duration in hospital exceeds 20 days following ascending aorta replacement, thus, it is indicated that a prolonged duration in hospital results in the reduction of postoperative ADL [14].

In recent years, the number of surgeries performed in the elderly has been increasing, thus, postoperative ADL can be extremely important in considering treatment strategy. ADL reduction is a serious problem in the elderly, and the risk of developing life-threatening complications, including aspiration pneumonia, may be increased. ADL reduction may be responsible for the lower cumulative survival rate in the TAR group in this study, although a significant difference was not observed. Therefore, debranching TEVAR may be a highly useful procedure for aortic arch aneurysm in the elderly, since shortening of the postoperative time in hospital can be expected in this procedure.

In discussion of the medium- and long-term results, no significant difference was observed in the aorta-related event rate between the d-TEVAR and TAR groups. A previous report has indicated a higher re-intervention rate after debranching TEVAR than after TAR [15]. While a higher re-intervention rate was also observed in the d-TEVAR group in this study, no aorta-related deaths were observed. As far as the elderly are concerned, overall survival was low because deaths in the elderly were caused by multiple diseases. Hence, a significant difference in aorta-related event rate, which is considered to be often observed after debranching TEVAR than after TAR, is unlikely to appear.

To perform endovascular treatment for thoracic aortic arch aneurysm, whether the procedure can be anatomically accepted is the absolute requirement, thus, it remains a prominent concern that a procedure cannot be applied to all patients. As debranching TEVAR without sternotomy was selected in this study, cases requiring an approach to the more proximal landing than zone 1 were not included in the d-TEVAR group. This may be a limitation of this study. Special procedures, including total debranching requiring sternotomy, chimney graft technique and fenestrated or branching type endograft, are necessary for treatment including zone 0. Previous studies have reported that these respective procedures were related with higher rate of complication like cerebral infarction, or higher rate of endoleaks, and, thus, their reliability still remains controversial [16]. However, as it is probable that treatment for thoracic aortic arch aneurysm in the elderly will benefit from endovascular treatment, future advancement of the devices is required.

## Conclusion

In consideration of the time required for the recovery of ADL, debranching TEVAR could be one of the choices for the treatment of aortic arch aneurysm in the elderly aged 75 years or older, especially with comorbidities.

### Limitations

Main limitations of this study are its retrospective nature, small sample size and being performed in a single-center. Although using a PS matching, definitive conclusion may be difficult to be drawn due to small number of patients studied. Further study with increased number of patients should be warranted.

## Data Availability

Supporting data are available upon request through the corresponding author. Publication of raw data is not possible as it will conflict with our privacy policy.

## Abbreviations

ADL: Activities of daily living
COPD: Chronic obstructive pulmonary disease
d-TEVAR: Thoracic endovascular treatment with debranching
LAA: The left axillary artery
LCCA: The left common carotid artery
PND: Permanent neurological deficit
PS: Propensity score
RAA: The right axillary artery
TAR: Total arch replacement through median sternotomy
TND: Transient neurological deficit
